# Eradication of mature biofilm from the isthmus region using Er, Cr: YSGG Laser as an activator of 2% chlorhexidine gluconate

**DOI:** 10.4317/jced.60964

**Published:** 2024-02-01

**Authors:** Ghufran I. Ibrahim, Hussein A. Jawad

**Affiliations:** 1 University of Baghdad, Institute of laser for postgraduate studies, Baghdad, Iraq; 2 Department of Biomedical Applications, Institute of Laser for Postgraduate Studies, University of Baghdad, Baghdad, Iraq

## Abstract

**Background:**

Challenges in the root canal system, such as isthmus, may limit the action of endodontic equipment and irrigation solutions, so using laser agitation is recommended to upgrade the removal of microbial biofilm. The objective of this study is to assess the effectiveness of the laser in photon-induced photoacoustic streaming protocol agitation of 2% chlorohexidine gluconate in removing mature biofilm in complex root canal systems.

**Material and Methods:**

Seventy-five mesial roots of the lower first and second molars were separated and cultivated with Enterococcus faecalis bacteria for 30 days (except for the negative control group samples). The samples were divided into four groups (n=15), one group acted as a positive control, other groups were irrigated with 2% chlorohexidine gluconate, some of them were agitated with a passive ultrasonic device, while the other samples were agitated by an Erbium, chromium-doped yttrium, scandium, gallium, and garnet laser in photon-induced photoacoustic streaming protocol. An atomic force microscope was used as a new method to get the results in the isthmus area; A scanning electron microscope was also used in the study to examine the samples before and after the treatment. Statistical Package for Social Sciences software was used to collect and analyze data, and two-way ANOVA was used to compare the means of the test groups.

**Results:**

According to the atomic force microscope and scanning electron microscope analyses, Erbium laser and passive ultrasonic activation groups showed higher antimicrobial efficacy than the syringe irrigation group (*p*<0.05).

**Conclusions:**

According to the study’s results, the agitation of chlorohexidine gluconate fluid by Erbium laser in photon-induced photoacoustic streaming at 0.75 W offers better Enterococcus faecalis biofilm removal in the difficult-to-reach areas of lower first and second molars.

** Key words:**Atomic force microscope, 2% chlorhexidine gluconate, Enterococcus faecalis, Erbium, chromium-doped yttrium, scandium, gallium, garnet laser, passive ultrasonic.

## Introduction

Bacterial and microbial pathogens in the root canal system are the principal pathogenic agents of dental pulp and periapical infections. *Enterococcus faecalis* (*E. faecalis*) is the most usually discovered bacteria following failed root canal therapy. *E. faecalis* is a gram-positive, facultative anaerobic bacteria that is particularly difficult to remove from challenging regions using standard procedures (1). Isthmuses act as a considerable challenge area for root canal cleaning and obturation. Their ribbon shaped, size, and extension laterally from the main canal make mechanical instrumentation impossible (2). So, the purpose of the irrigating solutions is to clean the canal system’s components that have missed or not reached by the mechanical instruments. Chlorhexidine gluconate (CHX) is a broad-spectrum antibacterial fluid that works to combat both gram-positive and gram-negative bacteria as well as yeasts, irrigating with 2 percent chlorhexidine gluconate was better than 0.12 percent (3). It was demonstrated that the effectiveness of using traditional chemo-mechanical approaches to access the entire root canal architecture is restricted, so different agitation strategies have been proposed to get around these restrictions. Erbium, chromium-doped yttrium, scandium, gallium, and garnet (Er, Cr: YSGG) is a type of water-absorbing laser with a wavelength of 2,780 nm (4). It has been suggested that the hydrokinetic energy employing promotes dental canal disinfection with no thermal harm to the underlying tissues (5). Recently, the Erbium laser family’s photon-induced photoacoustic streaming (PIPS) technology has demonstrated encouraging results in the elimination of biofilm from the root canal surface by irrigants agitation mechanism (6,7). Each impulse generated by the PIPS tip gets absorbed by the water particles, resulting in the production of a powerful “shock wave” that results in the creation of effective fluid streaming within the root canal system (8). In this investigation an atomic force microscopy (AFM) was used to evaluate biofilm removal, which is a useful technique for studying the shape and texture of various surfaces. Surface texture includes roughness, waviness, and flaws(9). This method has been widely utilized to investigate the mechanisms of antibacterial substance activity on bacteria (10). López-Jiménez *et al*. used AFM to examine the changes on the surface of *E. faecalis* after treatment with Er, Cr: YSGG laser, and Diode lasers and found that AFM is a good instrument that could be used to determine microbial viability as a measure of antimicrobial impact (11). Also, Kishen *et al*. investigated the effects of endodontic irrigation fluids on *E. faecalis* adherence to root canal dentin using AFM and discovered that employing endodontic disinfectants dramatically reduced *E. faecalis* adhesion to dentin (12). In 2020, Jiang *et al*. evaluated the bacterial eradication efficacy of CHX activated with a laser at the roots’ apical one-third and tested by using a 3D fluorescence microscope, they concluded that a greater number of bacteria were destroyed with laser-assisted irrigation immediately following treatment (13). This study aimed to investigate if agitating the root canals with an Erbium laser improved the efficiency of 2% CHX against mature E. faecalis biofilm in the lower human molar systems.

## Material and Methods

**Figure 1:**
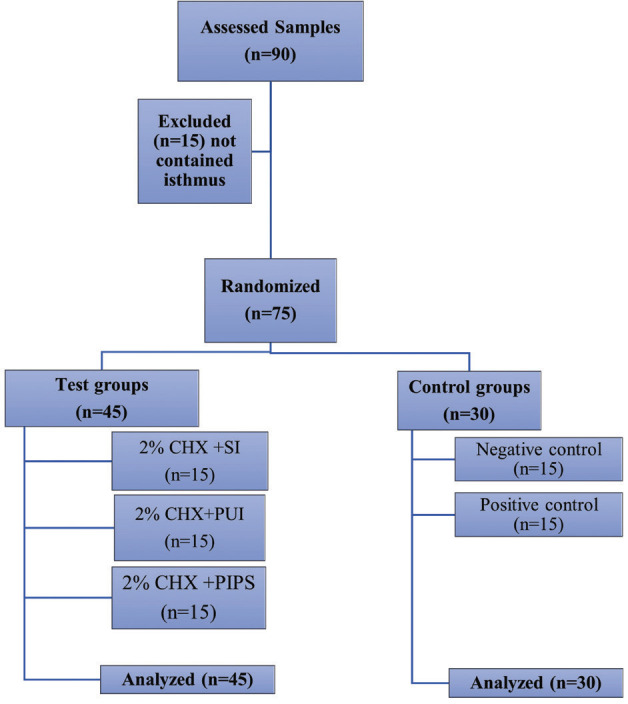
A flow diagram depicting the key stages of experimental protocol.

Ethical approval (102022-488) was obtained from the Research and Ethics Committee of Institute of Laser for Postgraduate Studies, University of Baghdad. The study design is illustrated by a flow diagram in Figure 1.

-Specimen Selection and Preparation

A total of 75 extracted mandibular first and second molars without root canal fillings, root caries, or restorations were obtained and cleaned immediately after extraction. Sample size calculation was done related to the following equation: n=((Z.σ)/E)2, where n=sample size, Z=standard score corresponding to the confidence level (0.05) which is (1.96), σ = The standard deviation of the variable, E: margin of error.

All samples were placed in a glass container containing distilled water with 0.1% thymol crystals (Lab Grade, Lab Alley, Texas, USA) until the day of the experiment.

A diamond disc (OSA-E28, Osakadent group Ltd., Guangdong, China) was used for cutting off the teeth crowns, and the mesial roots of all molar teeth were separated from the rest of the teeth to supply roots with a length of 12 mm from the apex. All separated mesial roots were imaged using cone-beam computed tomography (CBCT), samples that have a continuous isthmus between the mesiobuccal and mesiolingual canals were selected. A stainless-steel K-type hand file #10 (Dentsply, Maillefer, Ballaigues, Switzerland) was used to locate the site of the apical foramen. After sighting the file tip through the apical foramen, 1 mm was subtracted from the file length that was measured, and this value was used as the working length (WL).

All root canals were prepared to this WL up to #25/.04 NiTi engine files (X3 Never Break Serious, Easyinsmile, New Jersey, USA) at speed and torque recommended by the manufacturer. During instrumentation, 1 ml of 5.25% NaOCl (Cerkamed, Stalowa Wola, Poland) was administered after each file size, using a 30-gauge irrigation needle with side vents (Endo top, Hang Zhou Endo Top Medi-Tech Co. Ltd., Zhejiang, China). 1 ml of 17% EDTA was used as the final irrigate (Cerkamed, StalowaWola, Poland) and left in situ for 3 minutes. Within this period; the solution was activated with an ultrasonic device (Guilin Woodpecker Medical Instrument Co. Ltd, Guangxi, China) for 30 seconds.

At the last stage of the process, all the root samples were irrigated with 5 ml of distilled water (*Pi*oneer Company, Baghdad, Iraq) to eliminate the remains irrigation solutions. The root canals and the outer surfaces of the teeth were dried internally with paper points (Sure-endo, Sure Dent Corporation, Gyeonggi-do, Korea) and externally with paper towels. All roots were placed individually in Eppendorf tubes in an upright posture (Lab Serv, Thermo fisher Scientific, Gurugram, India) and autoclaved for 20 minutes at 121°C and 15 psi pressure.

-Bacterial Inoculation

Multiple *E. faecalis* colonies were taken from the agar plate (Himedia, Mumbai, India) and activated by being placed in brain heart infusion (BHI) broth (Himedia, Mumbai, India) a day before. Then 1 ml of bacterial infusion was diluted by adding 8 ml of normal saline to obtain a suspension equal to the McFarland standard (1.5 × 10 8 colony forming units CFU/ml). The suspension of bacteria was injected into the root canals (except of fifteen roots which act as a negative control group) using a disposable syringe and a 30-gauge irrigation needle until they were filled completely. Then all samples were immersed in 1.5 ml of BHI broth tubes and kept in an aerobic warm environment at 37°C for 30 days. Re-inoculations were carried out every three days to ensure the presence of live bacteria during the incubation period, and the BHI was replaced daily with a new one. All steps were performed in a sterile environment.

-Treatment groups

At the end of the incubation time, an injection needle was used to remove the liquid medium from the tubes, and each sample of the tested groups was subjected to multiple processes, including cleaning the samples’ surfaces with sterile cotton pads dipped in 5.25% NaOCl. The samples were then placed in plastic tubes filled with alginate impression material (Kromalgin, Vanninidental, Grassina, Italy) for simple handling, then randomly dividing into four groups:

(G A): The positive control group (n=15), the samples of this group did not get any sort of therapy.

(G B): 2% CHX +SI group (n=15), samples were irrigated with 2% CHX delivered by 30-gauge irrigation needle and kept the fluid inside the canals for 2 minutes, then washed with 5 ml of distilled water.

(G C): 2% CHX + passive ultrasonic activation (PUI) group (n=15), samples were also irrigated with 2% CHX fluid and left inside the canals for 2 min, then activated by a passive ultrasonic tip for 60 sec within the irrigation time then washed with 5 ml of distilled water.

(G D): 2% CHX + Er, Cr: YSGG laser at {60µs/pulse, 5 Hz, (0.25, 0.5, 0.75, 1, 1.25) W in PIPS protocol group (n=15), samples were irrigated with 2% CHX and left the fluid inside the canals for 2 min, within this time, the fluid was activated by 2780 nm Er, Cr: YSGG laser (Biolase, Waterlase, iPlus, CA, USA) for 60 sec. Laser operation proceeded for thirty seconds of “on” time, followed by thirty seconds of “off” time, and this sequence was repeated twice (for a total of 60 seconds of activation). After that, the canal was irrigated with a sterile solution of saline. Infrared laser safety glasses (Innovative Optics, Hemlock Lane North Maple Grove, USA) were worn before laser activation. A newly designed water Lase iPlus /MD glass tip was used (MZ6 Zip tip diameter = 600 μm, length = 6 mm). The laser unit’s water, as well as air spray were both set to ‘’off ‘’. During laser work, the tip was put just into the canal opening, remained stationary, and didn’t move apically into the root canal.

-Samples preparation for AFM and SEM examination

A properly fitting paper point cones were placed in the root canals to prevent tooth fragments from entering the endodontic canals and isthmus area. All roots were grooved longitudinally on their outer surface using a diamond disc, and a chisel was used to cut specimens in halves. Then, a middle area of the isthmus was marked for examination by the AFM (Nanosurf, Liestal, Switzerland).

The remaining halves of the roots were mounted on an aluminum base and metalized with a gold covering by vacuum evaporation, then a scanning electron microscope (SEM) (Inspect F-50, FEI Electron Optics International B.V., Netherlands) was used to examine the samples at 2 000 magnification power.

-Statistical analysis 

The data has been interpreted using the Statistical Package for Social Sciences (SPSS) version 21 (IBM, Armonk, New York, USA). It was provided as a mean, while categorical data was displayed using the standard deviation, data were expressed as a mean±SD. Two–way Analysis of variance (ANOVA) were utilized to compare the means of the tests. Post-hoc least significant difference (LSD) test was used to compute the significant differences among the tested means, and letters (a, b, c, d, e, and f) represented the levels of significant, highly significant start from the letter (a) and decreasing with the last one. Similar letters mean there are no significant differences between tested mean. Results of *p*>0.05 were considered statistically non-significant, while p≤0.05 was considered a significant value.

## Results

**Figure 2:**
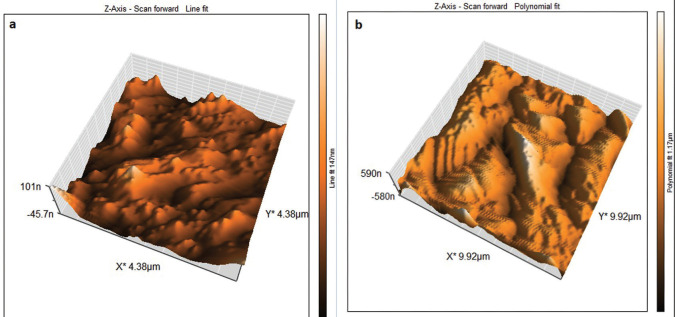
AFM three dimensions images about 6 mm from the apex of (a) Uncultivated isthmus surface (b) The isthmus surface after inoculated with E. faecalis for 30 days.

**Figure 3:**
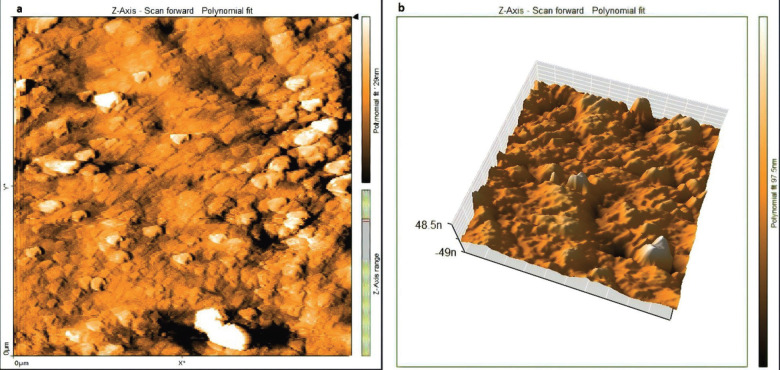
AFM analysis (a) Two and, (b) Three dimensions imaging of middle isthmus surface after treatment with 2% CHX that agitated by Er, Cr: YSGG laser at 0.75 W in PIPS protocol.

**Figure 4:**
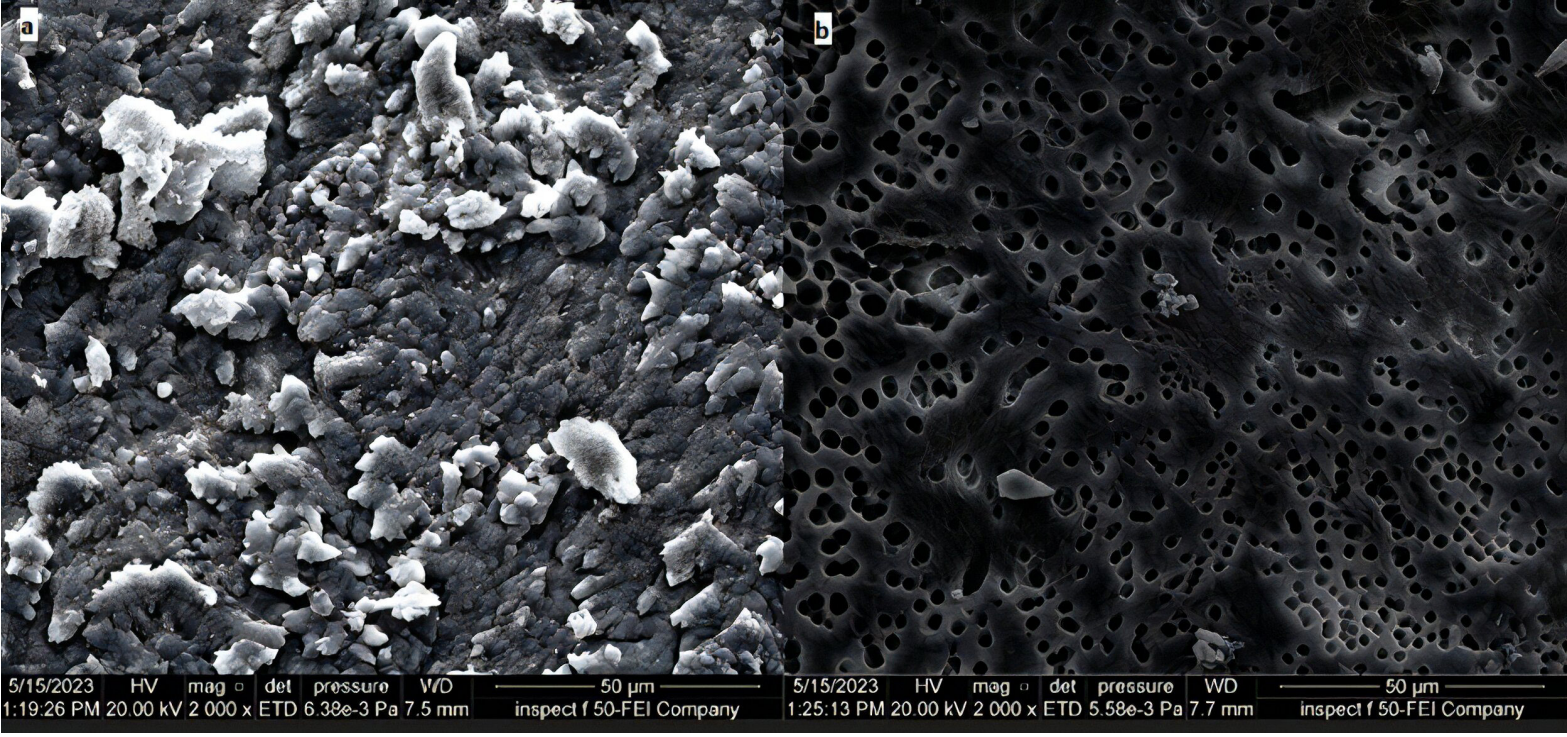
FE-SEM (2 000x) of the isthmus area about 6 mm from the apex (a) The positive control sample (b) The same area after being treated with 2% CHX+ Er, Cr: YSGG laser using PIPS at 0.75 W.

The effectiveness of laser agitation and other conventional techniques in the removal of bacterial biofilm in the isthmus was evaluated by using an AFM tool that measures the surface roughness to indicate the viability of the biofilm in the isthmus region. The uncultivated isthmus area was illustrated in the three-dimensional image (Fig. 2a), which shown relatively low peaks and shallow valleys. Also, 3D images of cultivated not treated isthmus surface was shown very high peaks and low valleys (Fig. 2b). In Figure 3, the images of the isthmus surface were exhibited after being treated with 2% CHX that agitated with Er, Cr: YSGG laser in PIPS at 0.75 W, showing a random or reduced spiky surface. The values of root mean square Sq., average roughness Sa., and maximum height Sz. of uncultivated isthmus area were 17.73±3.9, 13.60±2.3, and 136.5±2.05nm, respectively, as well as 567.6±26.2, 434.3±8.70, and 4050±6.55 nm, respectively, for cultivated samples. While parameters of the surface profile for the laser-agitated group samples at 60 µs, 5 Hz, and 0.75 W were 169.5±8.0, 135.1±8.79, and 1006±25.5 nm, respectively. The obtained Sq. value was illustrated in  Table 1 and showed that the lowest root mean square value was presented in the 2% CHX group that agitated by laser PIPS at 0.75 W (G D), followed by 2% CHX group that agitated by PUI (G C) and 2% CHX + SI group (G B). The highest root mean square value was presented in the positive control group (G A). To confirm the results obtained from an AFM test, the isthmus area was also evaluated by SEM taken at 6 mm from the apex at (2 000x) for a more detailed view of the biofilm and bacterial cocci. After bacterial incubation for 30 days, a dense and heavy layer of bacterial biofilm formed on the isthmus surface, occluding the dentinal tubules, as shown in Figure 4a. The samples treated with the laser PIPS at 0.75 W and 2% CHX showed a clean surface with open tubules and few smashed bacterial biofilms and debris remained on the surface, as shown in Figure 4b.

**Table 1:**
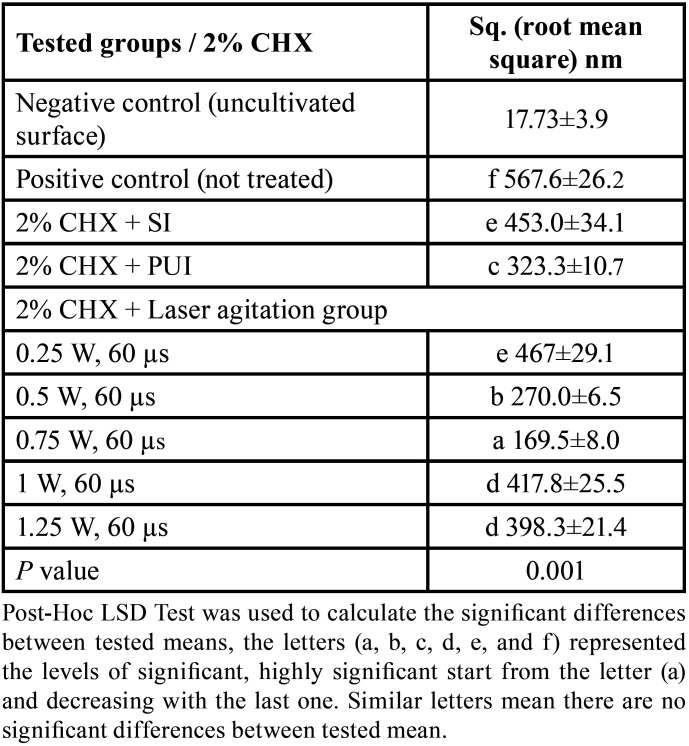
Means and standard deviations of root mean square values obtained from all samples, Post-Hoc LSD test performed after two-way ANOVA tests.

## Discussion

The primary goal of endodontic therapy is to completely clean the root canal, which is difficult due to the complex anatomy of the canal system. In past experiments, both culture procedures and molecular methods have been used to determine the number of viable bacteria in the canals and dentine tubules(14). A confocal laser microscope (CLSM) can also detect the viability of bacteria colonizing the root canal walls as well as the lateral canals and isthmus(15). This study used an atomic force microscopy (AFM) tool to analyze the samples, which is easy to apply, precise, and available. Surface roughness was determined by computing the parameters of the surface profile: root mean square (Sq.), average roughness (Sa), and maximum height (Sz.). The roughness parameters are determined via analyzing topographical scans of the sample’s isthmus surface. In this investigation, (sq.) was dependent, which is the root square of the surface height distribution, and is thought to be more responsive to significant deviations from the mean line than average roughness (9). Based on the obtained results, the computed root mean square value of the uncultivated isthmus surface was extremely low (17.73±3.9 nm), whereas the sq. of the surface following the growth of mature biofilm was significantly high (567.6±26.2 nm). The increase in surface roughness shows that biofilm growth resulted in a more textured and uneven surface. The measured sq. value of the samples that were treated with Er, Cr: YSGG laser at 0.75 W and 2% CHX (169.5±8.0nm) is significantly less than the values measured after other methods (PUI and SI) (323.3±10.7 and 453.0±34.1 nm), which means the reduction in *E. faecalis* biofilm on the isthmus surface by laser PIPS was more effective than other traditional methods. The PIPS technology works by absorbing laser photonic energy by the irrigation solutions that fill the root canals and without this absorption, the actual effectiveness of the laser does not appear (16). For this, the wavelength of the laser employed must match the absorption peak of the substance present inside the canals (17). As it is known, 2780 nm Er, Cr: YSGG lasers are well-absorbed by water chromophore (18), therefore high absorption of laser energy was ensured by the CHX fluid which is a watery solution. The shock wave generation is dependent on the absorbed energy of photons and the short pulse duration, which allows the energy to be focused for a short time and not dispersed to the surrounding areas, resulting in strong pressure and shockwaves that spread three-dimensionally within the root canals that are filled with liquids, allowing irrigant to reach difficult-to-access areas without placing the tip near the morphologically thinning apical third (19). The CHX irrigation solution is known to be limited in its effectiveness in removing biofilm (20), so the laser energy not only pushed the liquid to areas difficult to reach conventionally by generating a shock wave, but it also caused cavitation, which weakens the cell membrane and allows irrigants to penetrate bacteria’s cells (21). The SEM images demonstrated that a considerable portion of the isthmus surface and dentinal tubules were free of bacterial biofilm after treatment with the laser agitation approach. This affirms the results of the AFM investigation and demonstrates that laser agitation is quite successful in eliminating biofilm from these regions.

The obtained results were in good agreement with many previous studies, such as the study done by Sahar-Helft *et al*., which examined the actions of SI with 2% CHX and Er:YAG using LAI on *Enterococcus faecalis* and discovered that the total number of microorganisms dramatically decreased after being treated with LAI and CHX (22). Also, Aydin *et al*. compared the antimicrobial effects of 2.5% NaOCl and 2% CHX irrigation fluids activated with Er,Cr:YSGG laser (LAI) and an ultrasonic device on a 4-week-old *E. faecalis* biofilm, and concluded that activating NaOCl and CHX irrigation fluids with Er,Cr:YSGG pulsed laser or an ultrasonic technique can be useful in the eradication of *E. faecalis* bacteria from the canals (23). This investigation was constrained by the formation of microscopic fragments during the cutting stage, which could increase the value of surface roughness. However, this drawback was minimized with the use of a paper point placed in the canal to prevent debris from entering the isthmus during the cutting phase. Another drawback that might affect the outcomes is the anatomic variation of the isthmus area of the selected teeth.

## Conclusions

In the light of the results of this study, it is possible to conclude that Er, Cr: YSGG pulsed laser at 0.75 W in PIPS that activated CHX provide better biofilm removal in the difficult-reach isthmus region than other traditional irrigation methods.
